# Antibiotic Resistance Profiles of *Salmonella* Recovered From Finishing Pigs and Slaughter Facilities in Henan, China

**DOI:** 10.3389/fmicb.2019.01513

**Published:** 2019-07-04

**Authors:** Zenghai Jiang, Narayan Paudyal, Yaohui Xu, Tongwei Deng, Fang Li, Hang Pan, Xianqi Peng, Qigai He, Min Yue

**Affiliations:** ^1^ Division of Animal Infectious Diseases, State Key Laboratory of Agricultural Microbiology, College of Animal Sciences and Veterinary Medicine, Huazhong Agricultural University, Wuhan, China; ^2^ College of Veterinary Medicine, Henan University of Animal Husbandry and Economy, Zhengzhou, China; ^3^ College of Animal Sciences, Institute of Preventive Veterinary Medicine, Zhejiang University, Hangzhou, China; ^4^ Animal Health Research Division (AHRD), Nepal Agricultural Research Council (NARC), Kathmandu, Nepal; ^5^ Zhejiang Provincial Key Laboratory of Preventive Veterinary Medicine, Hangzhou, China

**Keywords:** *Salmonella*, multidrug resistance, China, quinolones, beta-lactamase, prevalence

## Abstract

With the increase in commercial pig farming, there is a simultaneous increase in the use of antibiotics for prophylaxis as well as therapeutics in China. In this study, we evaluated the prevalence and resistance diversity of salmonellae isolated from feces of asymptomatic, live and slaughtered pigs. We analyzed 1,732 pig fecal samples collected over 8 months, at Henan province of China. The salmonellae were isolated and identified by PCR. They were serotyped using commercial antisera and assayed for the MIC of 16 antibiotics by broth microdilution method. The average prevalence of *Salmonella* was 19.4% (95% CI: 17.6–21.4). Large farms (herd size ≥1,000) were found to have a higher prevalence as compared to the small- and medium-scale farms (*p* < 0.0001). The prevalence of salmonellae in samples collected from the farms [11.77% (95% CI: 10.1–13.6)] and from the slaughterhouse [45.23% (95% CI: 40.3–50.30)] was statistically different (*p* < 0.0001). Uncommon serovars of *Salmonella* such as Agama and common serovars such as Derby and Typhimurium were isolated. High resistance (>80%) was recorded toward ciprofloxacin (100%), tetracycline (99.4%), doxycycline (97%), sulfamethoxazole (85.8%), ampicillin (81.6%), and amoxicillin (80.4%). Multidrug resistance (MDR) to four, five, and seven classes of antibiotics was recorded to be approximately 25% in the most prevalent serovar like Derby. We conclude that the presence of alarmingly high resistance, toward the critical antibiotics such as fluoroquinolones and beta-lactams, in large swine farms in China, should draw public attention. These results highlight the need for continued antibiotic stewardship programs for judicious use of critical antibiotics in animal health as well as for producing safe pork.

## Introduction

Fuelled by the rapid socio-economic growth and urbanization of China, the average intake of meat, especially pork, increased from 37.1 g/day in 1992 to 64.3 g/day per person in 2012 (He et al., 2016). With the population growth projected to reach 1.4 billion in 2020, the consumption of pork is also expected to surpass 100 g/day per person by 2020 (CIIN, 2018a). In 2017, Henan province alone contributed over 65 million live pigs, approximately 20% of the whole national consumption (CIIN, 2018b). The boost in pig production and consumption in China has indirectly increased the risk of foodborne zoonoses, including salmonellosis. Previous studies have shown that *Salmonella* is one of the leading foodborne pathogens on food commodities, meat in particular, and play a significant role for causing human diarrhea in China and elsewhere (Pan et al., 2018, 2019; Paudyal et al., 2018).

Routine surveillance of potential hazards is essential to minimize the risks of disease epidemics as well as other potential threats such as pathogens and antibiotic resistance (AR). The finishing herds usually ready for slaughter require special attention in the surveillance purpose because they could disseminate the pathogens, if any they carry in the gut. Studies in other parts of the world have investigated the carriage of salmonellae in apparently healthy, asymptomatic pigs in the finishing herds (Baptista et al., 2009, 2010, 2011; Wilhelm et al., 2012). There are numerous studies about antibiotic-resistant salmonellae in pigs in China. The prevalence of AR reported in these studies varies greatly but generally is reported toward critical drugs, including quinolones and cefotaxime (Bai et al., 2016), tetracyclines (Jiu et al., 2017), quinolones, and cephalosporins (Jiang et al., 2014) or toward trimethoprim-sulfamethoxazole, ampicillin, and tetracycline (Su et al., 2018).

Pathogens and AR can arise from multiple sources, for example, the endemic pathogens circulating in the farm or pathogens introduced through the feed, water, workers, and equipment. Slaughterhouse is one of the most important risk factors that can act as a mixing vessel for any kinds and numbers of pathogens including *Salmonella* present in animals collected from different unrelated farms. Under favorable circumstances, such pathogens can be disseminated *via* the meat to the consumers.

Previous studies on similar topics are essential for understanding the scope and magnitude of salmonellae prevalence, as well as the corresponding AR crisis in China. However, most of those studies have focused on clinically diseased pigs or retail-meat/pork. The overall burden of foodborne pathogens, including *Salmonella*, plays a significant threat to food safety and public health. Because Henan contributes a major segment of pork production in the Chinese national scenario, it is necessary to assess and inform the stakeholders on the possible prevalence of zoonotic pathogens like *Salmonella* recovered from swine finishing herds and slaughter facility. Additionally, co-existence of different models of farming systems, for example, small households manual farms with less than 100 animals per farm to large intensive automated commercial farms with more than 10,000 animals per farm, demand different strategies to prevent and curtail the prevalence of pathogens like *Salmonella* and AR. With an aim of minimizing these knowledge gaps, we examined the distribution, prevalence, and AR patterns of *Salmonella enterica* recovered from farms, and slaughterhouses in Henan province, one of the Chinese leading pig/pork producers.

## Materials and Methods

This study was carried out in a cross-sectional observational design, to estimate the prevalence of various serovars of salmonellae in pig feces. The quantification of the minimum inhibitory concentration (MIC) of 16 molecules belonging to nine different classes of antibiotic agents was conducted simultaneously.

### Sample Collection

From March 2017 to November 2017, a total of 1,732 fecal samples (approximately 100 g per animal) were collected from randomly selected 36 small- and medium-scale farms (SMS-farms, housing <1,000 head of pigs), nine large-scale farms (housing ≥1,000 head of pigs), and two pig slaughtering facilities at Henan province in China (slaughtering capacity of 3,000 heads of pigs/day). In pig farms, 1,334 fresh fecal samples (499 from large-scale farms and 835 from SMS-farms) were collected from apparently healthy or asymptomatic finishing pigs by non-invasive sampling technique. In addition, a total of 398 rectal fecal samples were collected from two slaughterhouse facilities. The samples were collected aseptically and processed on the same day of collection.

### Isolation and Identification

The primary culture and isolation of the organism were done according to the protocol recommended by the World Organization for Animal Health Terrestrial Manual (OIE, 2016). This method consists of pre-enrichment in buffered peptone water (BPW) followed by enrichment in modified semisolid Rappaport Vassiliadis (MSRV) plate and final isolation on xylose lysine deoxycholate (XLD) agar plate. The obtained isolates were then confirmed by polymerase chain reaction (PCR). To elaborate, 10 g of feces were added to 90 ml of BPW and incubated at 37°C for 18–20 h. Three hundred microliters of thus incubated BPW was transferred to MSRV semisolid agar plate and incubated at 41.5°C for 24 h. An opaque halo in the MSRV medium is indicative of growth. A 10 μl loopful of the bacterial growth from MRSV medium was transferred to XLD agar plates using disposable sterile inoculating loops and these plates were incubated further for 18–20 h at 37°C. Typical round red colonies with black center on XLD media were considered presumptive of salmonellae. Other variant forms such as transparent single colonies, transparent pink colonies or transparent colorless colonies were sub-cultured on Brilliant Green Agar plates for two more times until they were declared negative and discarded. We picked up three isolated pure colonies for each sample and sub-cultured them for any mixed populations. Upon obtaining homogeneous colonies in all the three plates, only one pure isolated colony from the plate number one, was taken and purified further with one more passage in XLD plates. The pure colonies were then seeded in Luria-Bertani (LB) broth for serotyping and DNA extraction by boiling method. PCR using the primers for amplification of the enterotoxin *stn* gene for the confirmation of the *Salmonella* was performed as recommended (Zhu et al., 2015).

### Serotyping

The PCR confirmed salmonellae were serotyped according to White-Kauffmann-Le Minor scheme by slide agglutination to define O and H antigens using commercial antisera (S & A Reagents Lab, Bangkok, Thailand). Strains that did not express phase 2 flagellar antigen even after three attempts of phase inversion were considered monophasic.

### Antibiotic Susceptibility Test

**Figure 1:**
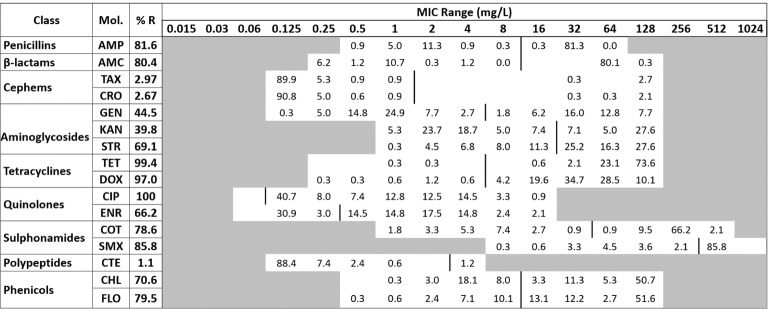
The distribution of the various minimum inhibitory concentration (MIC) levels of the *Salmonella* against 16 antibiotics. The names of the antibiotics (second column) are abbreviated as ampicillin (AMP), amoxicillin (AMC), cefotaxime (TAX), ceftriaxone (CRO), gentamicin (GEN), kanamycin (KAN), streptomycin (STR), tetracycline (TET), doxycycline (DOX), ciprofloxacin (CIP), enrofloxacin (ENR), sulfa-trimethoprim (COT), sulfamethoxazole (SMX), colistin (CTE), chloramphenicol (CHL), and florfenicol (FLO). The third column shows the average resistance (in percentage); the second row is the range of the MIC tested; and the values in each cell show the percentage of strains in that particular MIC dilution level. The vertical bar indicates the cutoff level of the minimum inhibitory concentration for each antibiotic at the highest value of that particular cell’s dilution for susceptibility (equal to or less than) and resistance (greater than). For the sake of clarity and facilitate analysis, the intermediate category was merged with the resistant.

The *Salmonella* isolates were subjected to antibiotic MIC assay using the broth microdilution method following the recommended procedure (CLSI, 2012). Sixteen different molecules of antibiotics belonging to nine classes were used for the MIC assay. The cutoff values recommended by CLSI (CLSI, 2016) except for enrofloxacin (Hao et al., 2013) and colistin (EUCAST, 2019) were used for the categorization of the results (Figure 1). The intermediate category was merged with the resistant ones for the analysis of our data. The classes of antibiotics and the MIC range (mg/L) used in our assay included penicillin (ampicillin or AMP, 0.5–64); beta-lactams (amoxicillin or AMC, 0.25–128); cephems (cefotaxime or TAX, 0.125–128; ceftriaxone or CRO, 0.125–128); aminoglycosides (gentamicin or GEN, 0.125–128; kanamycin or KAN 1–128; streptomycin or STR, 1–128); tetracyclines (tetracycline or TET, 0.25–128; doxycycline or DOX, 0.25–128); quinolones (ciprofloxacin or CIP, 0.06–16; enrofloxacin or ENR, 0.06–16); sulphonamides (cotrimoxazole or COT, 1–512; sulfamethoxazole or SMX, 8–1,024); polypeptide (colistin or CTE, 0.125–4); and phenicols (chloramphenicol or CHL, 0.5–128; florfenicol or FLO, 0.5–128).

### Data Analysis

The obtained numerical data were analyzed using Student’s *t*-test and ANOVA in GraphPad Prism vs 7 for Windows platform. The alpha (*p*) level of less than 0.05 was considered significant and they are given as exact values. The difference in the prevalence of the two types of farms (SMS-farms and L-farms) was compared using a two-sample independent *t*-test. A Chi-square test was used to compare the prevalence among the L-farms, SMS-farms, and the slaughterhouse. The difference in the cumulative prevalence of resistance among the salmonellae, isolated from L-farms, SMS-farms, and the slaughterhouse with respect to the individual antibiotic agent was compared using two-way ordinary ANOVA.

## Results

### Prevalence and Serovar Distribution

**Figure 2:**
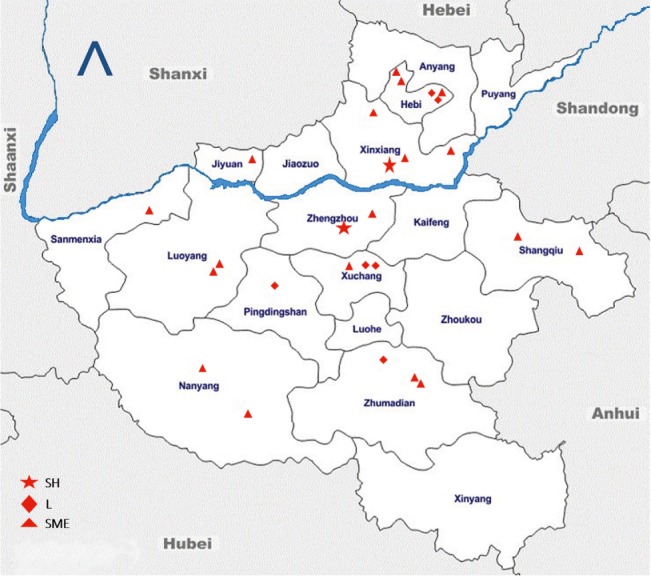
Geographic distribution of the sampling sites across different administrative divisions (or cities) in Henan, China. Sampling sites are denoted as slaughterhouse (SH), large farms (L), and small and medium enterprise farms (SME). The blue line denotes the Huang He river, flowing through the province to the east into the Bohai Sea. The arrow head points to the geographical north. This map is not drawn to scale.

During the sampling period of 8 months (March to November 2017), a total of 1,732 fecal samples (398 from the rectum of slaughtered pigs and 1,334 from live pigs) were collected and analyzed. The spatial distribution of these sampling sites is shown in the geographical map of Henan in Figure 2. This distribution shows that our sampling frame covered a larger geographical area within Henan; hence, we believe that these results are representative of the total population. A total of 337 samples (180 rectal samples collected at the slaughterhouse, 87 samples from large-scale farms, and 70 from SMS-farms’ fresh fecal samples) tested positive for *Salmonella*. The average prevalence of *Salmonella* was 19.4% (95% CI: 17.6–21.4) in this study (337 positives out of 1,732 samples, Table 1). The isolates belonged to 10 different types of serovars.

**Table 1:** Distribution of samples and various serovars at different sampling sites.

Site	Total samples	Serovars	Positive samples	%Prev. (FL)	%Prev. (SL)	%Prev. (Ov.)
L-farms		Agama	1	0.20	0.07	0.06
		Agona	2	0.40	0.15	0.12
		Derby	24	4.81	1.80	8.14
		Newlands	1	0.20	0.07	0.81
		Rissen	24	4.81	1.80	2.94
		Typhimurium	35	7.01	2.62	4.56
Subtotal L-farms [A]	499		87	**17.43**	**6.52**	
SMS-farm		Bovismorbificans	9	1.08	0.67	0.64
		Derby	29	3.47	2.17	
		Emek	2	0.24	0.15	0.17
		Newlands	4	0.48	0.30	
		Rissen	7	0.84	0.52	
		Typhimurium	19	2.28	1.42	
Subtotal SMS-farms [B]	835		70	**8.38**	**5.25**	
**Total farms [A + B]**	**1,334**		**157**		**11.77**	
SH		Bovismorbificans	2		0.50	
		Chester	2		0.50	0.12
		Derby	88		22.11	
		Emek	1		0.25	
		London	33		8.29	1.91
		Newlands	9		2.26	
		Rissen	20		5.03	
		Typhimurium	25		6.28	
**Total SH [C]**	**398**		**180**		**45.23**	
**Grand total [A + B + C]**	**1,732**		**337**			**19.46**

SH, slaughterhouse; Prev., prevalence; FL, farm level; SL, site level; Ov., overall.

The difference in the prevalence of salmonellae isolates from the fecal samples collected at the farms [11.77, 95% CI: 10.1–13.6 (*n* = 157, *N* = 1,334)] and at slaughterhouse [45.23 95% CI: 40.3–50.30 (*n* = 180, *N* = 398)] was compared using the two-sample independent *t*-test. The analysis showed that the higher prevalence in slaughterhouse samples was statistically significant (*p* < 0.0001). Among the 45 farms included in the study, nine farms were large commercial farms (L-farms) with ≥1,000 heads of live pigs while 36 were small- and medium-scale farms (SMS-farms) with <1,000 heads of live pigs. The prevalence of salmonellae in L-farms was 17.43% (95% CI: 14.2–21.1), whereas in SMS-farms, it was 8.38% (95% CI: 6.6–10.5). The results showed that the higher prevalence in L-farms is statistically highly significant (*p* < 0.0001). The comparison of prevalence among the L-farms, SMS-farms, and the slaughterhouses revealed that the difference in prevalence among these was statistically significant (*p* < 0.0001).

**Figure 3:**
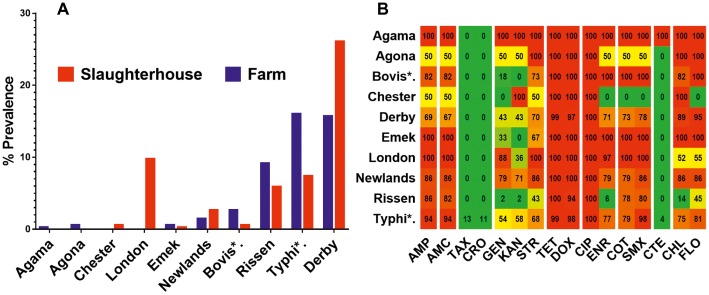
The serovar and antibiotic resistance diversity of *Salmonella* across farm and slaughterhouse. **(A)** Spatial variation of the distribution of multiple salmonellae serovars isolated from fecal samples analyzed in this study. **(B)** Distribution of the average resistance (in percent) of various serovars (YY′) irrespective of the source of isolation toward multiple antibiotics (XX′). The color scale of the individual cells is quantified by the numerical values in the heat map to indicate the resistance percentage. Abbreviated names of the serovars are Bovis*: Bovismorbificans and Typhi*: Typhimurium. The names of the antibiotics (XX′) are abbreviated as ampicillin (AMP), amoxicillin (AMC), cefotaxime (TAX), ceftriaxone (CRO), gentamicin (GEN), kanamycin (KAN), streptomycin (STR), tetracycline (TET), doxycycline (DOX), ciprofloxacin (CIP), enrofloxacin (ENR), sulfa-trimethoprim (COT), sulfamethoxazole (SMX), colistin (CTE), chloramphenicol (CHL), and florfenicol (FLO).

We differentiated 10 various serotypes of salmonellae in the total 337 positive isolates. The distribution of these serovars at different sampling sites is given in Table 1. Among the total samples collected (*n* = 1,732), Derby was the most prevalent serovar (8.14%, *n* = 141) while the least prevalent one was serovar Agama (0.06%, *n* = 1). Serovars such as Chester and London were isolated only from the slaughterhouse samples, while serovars like Agama and Agona, were isolated only from the farm samples. Serovars Bovismorbificans, Derby, Emek, Newlands, Rissen, and Typhimurium were isolated from both sample types. Among the total positive isolates (*n* = 337), serovar Derby was the most prevalent (26.1%, *n* = 88) in the samples from the slaughterhouse, Typhimurium was more prevalent (16%, *n* = 54) in the farms’ samples. The spatial (farms and slaughterhouses) variation in the distribution of prevalence of these serovars is given in Figure 3A.

### Antibiotic Resistance and Multidrug Resistance Pattern

**Figure 4:**
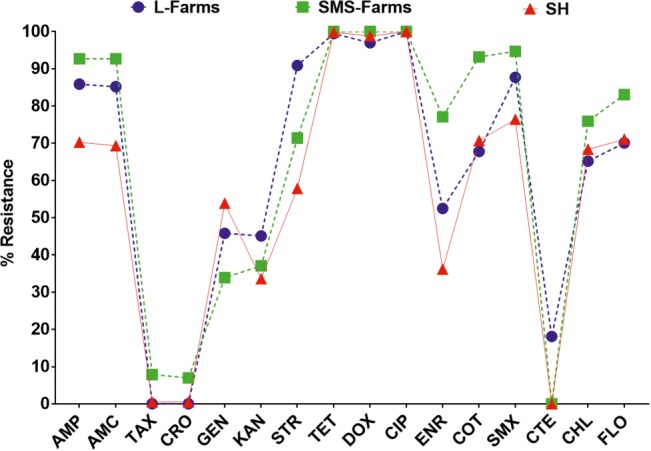
Distribution of the average resistance (in percent) of individual antibiotics in *Salmonella* isolates from samples collected across large farms (L-farms), small- and medium-scale farms (SMS-Farms), and slaughterhouses (SH).

Broth microdilution MIC assay was performed for 16 antibiotic agents of nine different classes. The prevalence of resistance among *Salmonella* isolates against the different classes of antibiotics is shown in Figure 3B. Notable was the presence of high resistance toward some clinically important antibiotics such as fluoroquinolones and beta-lactams. Low resistance rates (<3%) against cephems and colistin were recorded. The overall distribution of the MIC values for these antibiotics is shown in the squashtogram in Figure 1. The results showed that this difference in the overall average resistance among the sampling sites (L-farms, SMS-farms or SH) was statistically significant (*p* = 0.0128), whereas the difference in resistance to individual antibiotic agents was statistically highly significant, *p* < 0.0001 (Figure 4).

**Figure 5:**
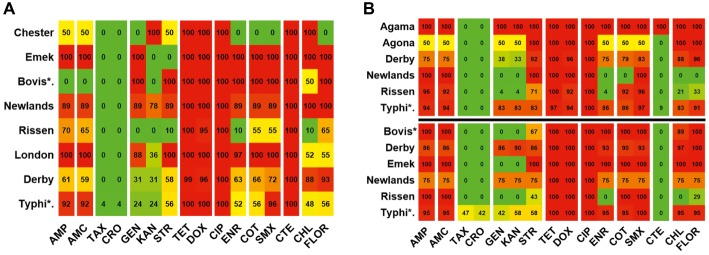
Antibiogram of the slaughterhouse and farms isolates. **(A)** Resistance distribution in the slaughterhouse serovars. The difference in the resistance of serovars is statistically significant (*p* = 0.0010), while the difference among the individual antibiotics is highly significant (*p* < 0.0001). **(B)** Resistance distribution in the farm serovars. The upper half (above the black horizontal line) is the distribution in the large-scale farms while the lower half (below the black horizontal line) is the distribution in small- and medium-scale farms. The variation of the resistance among the serovars was statistically non-significant (*p* = 0.2984) in either type of the farms, whereas the variation between the individual antibiotics was statistically significant (*p* < 0.0001). The color scale of the individual cells is quantified by the numerical value given within the cells. Abbreviated names of the serovars are Bovis*: Bovismorbificans and Typhi*: Typhimurium.

The resistance among the serovars isolated from the slaughterhouse samples was compared for further analysis. It was seen that while the resistance among the serovars was statistically significant (*p* = 0.001), the difference among the antibiotics was highly significant (*p* < 0.0001) (Figure 5A). When the resistance between two types of farms were compared, variation in the resistance among the serovars was statistically non-significant (*p* = 0.2984), whereas the variation between the individual antibiotics was statistically highly significant (*p* < 0.0001) (Figure 5B).

**Figure 6:**
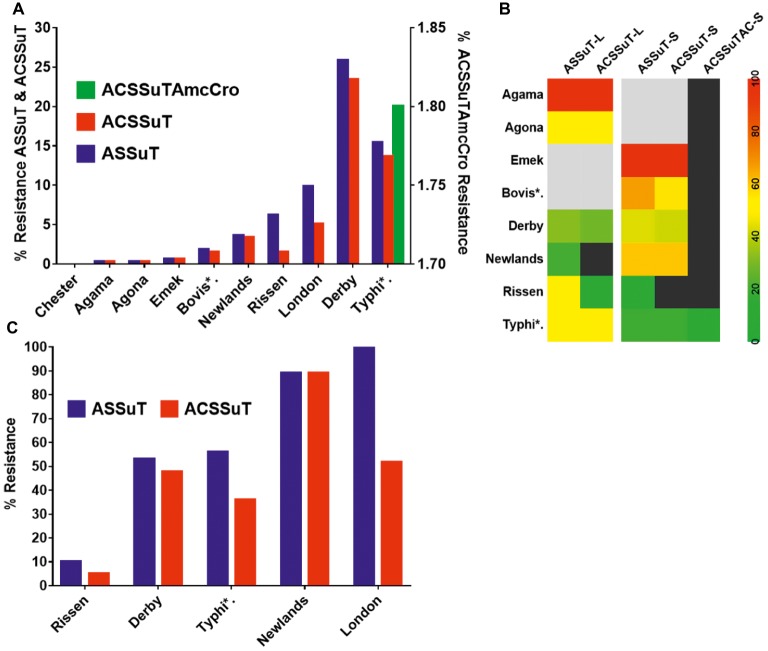
The multiple-drug resistance (MDR) patterns of *Salmonella* serovars. **(A)** MDR patterns for individual serovars isolated in this study. The left X-axis shows tetra- and penta-resistance while the right X-axis shows hepta-resistance pattern (seen only in serovar Typhimurium). **(B)** MDR pattern (in percent) according to the farm type. Gray boxes indicate the absence of serovars whereas black boxes represent the absence of resistance. L- indicates large farms, SMS- indicates small- and medium-scale farms. **(C)** MDR pattern in the isolates from the slaughterhouse. The pattern is abbreviated as resistance to A, Ampicillin; C, chloramphenicol; S, streptomycin; Su, sulfamethoxazole; T, tetracycline; Amc, amoxicillin, and Cro, ceftriaxone. Abbreviated names of the serovars are Bovis*: Bovismorbificans and Typhi*: Typhimurium.

The tetra-, penta-, and hepta-drug resistance patterns were analyzed for the isolates shown in Figure 6A. Tetra-resistance pattern (ASSuT, i.e., resistance to ampicillin, streptomycin, sulfamethoxazole, and tetracycline) was the most frequently (maximum of 25.8% for Derby) seen among the serovars. Penta-drug resistance (ACSSuT, i.e., ASSuT with chloramphenicol) was highest (23.4%) for serovar Derby. Hepta-drug resistance (i.e., ACSSuT with amoxicillin and ceftriaxone) was recorded only in serovar Typhimurium (1.8%).

The multidrug resistance (MDR) patterns were also compared in relation to the origin of the isolates (L-farms, SMS-farms, and slaughterhouses). The tetra- and penta-drug resistance patterns were high (>50%) in L-farms for Typhimurium and high (43%) in SMS-farms for Derby. Uncommon serovars like Agama (only on L-farms) and Emek (only on SMS-farms) were 100% MDR (Figure 6B). The prevalence of tetra- and penta-resistance patterns in the serovars isolated from slaughterhouse was more than 40% except in serovar Rissen (Figure 6C).

## Discussion

China produces and consumes more than half (at about 500 million heads per annum) of the global pork demand (Larson, 2015). Henan is one of the largest pig producing provinces of China. There are more than 3,000 farms (rearing 100–10,000 heads of pigs) contributing almost 62% of the production, while approximately 583 farms with a herd size of more than 10,000 contribute around 18% of the production (CIIN, 2018b). Pigs and pork from Henan are also distributed to other neighboring provinces like Zhejiang, Shanghai, Anhui, and Shanxi or to the south as far as Guangdong. These distribution channels also serve as a conduit for the transmission of pathogens as well as antibiotic-resistant strains from the farms to the distributors and the consumers. Thus, the prevalence of the pathogen in Henan pig/pork not only has a local but also widespread regional implication. Numerous earlier studies have reported on the spatiotemporal variation of the prevalence of salmonellae on different types of meat, foods, and animals across China (Yan et al., 2010; Li et al., 2013, 2014; Lai et al., 2014; Bai et al., 2015; Kuang et al., 2015; Cai et al., 2016; Jiu et al., 2017; Xu et al., 2017; Yi et al., 2017; Su et al., 2018). These studies suggested that pathogenic bacteria from animals can transmit AR determinants to strains isolated from humans.

In this study, the prevalence of the serovar Derby was the highest (41%, 141/337). An earlier study from Henan which analyzed eviscerated pig carcass swab samples reported that *S*. Typhimurium was the most prevalent serovar followed by Derby (Bai et al., 2015), whereas another study showed that Derby was the most prevalent serovars in pork (Zhu et al., 2019). For a comparison, serovar Typhimurium ranked first and Derby ranked the third most frequent cause of non-typhoidal salmonellosis in human patients in Henan (Xia et al., 2009). There are no reports on the presence of serovars such as Agama and Chester in Henan pigs, until date. Serovar Derby has also been reported to have the highest prevalence in the pig/pork from Jiangsu (Li et al., 2014) or from Sichuan (Li et al., 2013). Jiangsu and Henan are the neighboring provinces and there is the trade of pig/pork between these two places. Sichuan is one of the top producers of pig/pork in China. It has multiple inter-provincial trades, which are likely linked to each other along the pork value chain.

The prevalence of salmonellae was higher in large farms as compared to smaller farms and the difference was statistically significant. Common knowledge dictates that larger farms have stronger biosecurity measures and generally implement good farming practices, which should have minimized the prevalence of pathogens. However, the farm level prevalence is a multi-factorial outcome and factors such as stocking density, endemic contamination of the farm, histories of infection in the past, and flawed biosecurity measures could all be responsible for this increased prevalence. Antibiotics are frequently used to act as insurance to prevent a disease epidemic that is associated with overcrowding and poor sanitation in pig production. This can lead to lower apparent prevalence but probable longer shedding stage (Patterson et al., 2016) and increased resistance in small farms, which is evident in our analysis. The situation is further exacerbated because some antibiotics are widely used as a feed supplement primarily for growth stimulation. This could be the prime reason for the presence of almost similar prevalence rates of resistance to multiple antibiotics in the SMS- and large farms.

In our analysis, samples collected from the rectum of the slaughtered pigs had a higher prevalence of salmonellae as compared to the fresh fecal samples collected on the farm. The slaughterhouses from where the samples were collected for this study, provided service to multiple farms (even those beyond our sampling frame) throughout Henan. It was reported earlier that in apparently healthy flocks harboring *Salmonella*, 5–30% of the animals may still excrete the bacteria at the end of the finishing period which could probably double during transport and lairage (Berends et al., 1996). Stress due to transportation, along with a number of factors like environmental contamination and dose-response parameters, is mentioned to affect the pathogen secretion during lairage and subsequent slaughter (Simons et al., 2016).

The presence of less common serovars such as Agama (*n* = 1), Agona (*n* = 2), and Emek (*n* = 3) in our study shows that pigs not only contribute specific pathogenic serovars such as Typhimurium or Derby to humans but also harbor atypical strains. These atypical strains also showed higher levels of resistance for most of the common antibiotics used in pig husbandry in Henan. This is of concern because atypical strains, which are apparently harmless to pigs or humans, under the selective pressure of different antibiotics used in pig husbandry could play a significant role in the transmission of resistance to other pathogenic strains. Increase in AMR genes under selective pressure in swine fed with medicated feed has been previously reported (Looft et al., 2012). Currently, there are more than 20 classes of antibiotics with over 100 generic molecules but not all are equally used in animal husbandry (Zhang et al., 2015). Antibiotics are usually incorporated in animal feed at low concentrations (2.5–125 mg/kg) as growth promoters for an undefined time period (Marshall and Levy, 2011). An earlier report on a survey of 60 pig farms of various sizes during 2007–2008 in Henan province suggested that antibiotics were used in all stages of pig production, from weaning, to growing and fattening, as additives in animal feed, *via* drinking water or individual injection. The major classes of antibiotics used were tetracyclines, penicillins, sulfonamides, ceftiofur, and florfenicol (Zhang et al., 2015; Wu, 2018).

The occurrence of tetra- or penta-drug resistance in high frequency in the serovars Derby and Typhimurium decrease the chances of success of therapeutics at the farm level. The carriage of these MDR strains (ASSuT, ACSSuT, or ACSSuTAmcCro) by the pigs could potentially contaminate the downstream along the slaughter-chain. As the Henan pigs/pork are distributed over wider geography in China, this can eventually lead to a spread of the resistance strains throughout the pig/pork food chain.

Our results showed that the effect of the sampling site had a significant influence on the prevalence of resistant bacteria. A study from southern China reported that in typical large-scale commercial pig farms, the most frequently used “in feed” antibiotics were tetracyclines, bacitracin, or sulfonamides (Zhou et al., 2013). Yet another analysis from 2015 reported that in the various regions of China, amoxicillin had the largest use in animals as well as in humans, whereas florfenicol and quinolones were the most common antibiotics used in pig farms (Zhang et al., 2015). We can, therefore, infer that there is a high selection pressure of antibiotics in the swine gut thereby causing the appearance of high resistance to common “in feed” or therapeutic antibiotics such as sulfa-trimethoprim, tetracyclines, amoxicillin, ampicillin, and chloramphenicol. The antibiotic use in animal production is a key contributor to the growth of animal production to ensuring food security in China over the last four decades (Wu, 2018). This usage rate has also increased with the expansion of animal production and with more intensive livestock production. This has inadvertently led to an increase in the risk of zoonotic pathogens such as *Salmonella* with high MDR capable of emergence and dissemination *via* the food systems.

Unlike earlier studies that reported the presence of increasing levels of resistance by salmonellae to cephems class of antibiotics (Jiu et al., 2017), the strains in our collection were highly susceptible to this class of antibiotic. This finding is in accordance with regional differences and variations in the selection pressure on different farms (Zhou et al., 2013; Collignon and Voss, 2015). The resistance of atypical strains such as Agama to all other classes of antibiotics except the cephems is also an interesting finding in the sense that this serovar is not generally associated with mammals but rather with reptiles and amphibians. Its presence and the general resistance is a matter of concern and further assessments.

## Conclusion

The prevalence of salmonellae isolates differed significantly according to the farm size, with large farms having more salmonellae as compared to the small farms with the maximum prevalence in the slaughterhouse samples. The presence of high level of resistance to critical antibiotics such as quinolones and beta-lactams in the various serovars of salmonellae isolated from apparently healthy/slaughtered pigs possibly illustrates the risk of acquisition and dissemination of such MDR *via* the pork food chain. Presence of highly MDR atypical serovars exacerbates this risk even further as they might be acting as the “dark matters” for the resistance (Paudyal and Yue, 2019). This could eventually lead to therapeutic failures in animals as well as humans even for the clinically critical antibiotics.

## Ethics Statement

No ethical approval was deemed necessary for this study. Oral agreement and permission were obtained from the farmers as well as the slaughterhouse manager before sampling.

## Author Contributions

ZJ, YX, and TD collected the samples. ZJ, NP, FL, and HP did the lab analysis. NP, XP, and QH did the data analysis and prepared a draft. MY conceived the project and provided critical comments for the draft. All the authors have read and agreed to the manuscript.

### Conflict of Interest Statement

The authors declare that the research was conducted in the absence of any commercial or financial relationships that could be construed as a potential conflict of interest.
